# G. H. Q. Bulletin No. 54 on the Venereal Problem

**Published:** 1919-01

**Authors:** 


### G. H. Q. BULLETIN No. 54 ON THE VENEREAL PROBLEM

It can be said without fear of contradiction that the morals of the soldiers of the American Army are better than those of any army in the history of warfare. An annual venereal rate of 29 per thousand in the A. E. F., and 20 per thousand in the training camps in the United States, is unprecedented among soldiers, and so much better than that of the men who were drafted into the United States Army that the Regular Army surgeons speak of the " depravity of civil life ".

The record that has been made in the remarkable reduction of venereal diseases in the Army has been brought about by the efforts of the Medical Department of the Army with the active co-operation of the General Staff, and the line officers who have control of the discipline of troops. Too much credit, however, cannot be given to the Commission on Training camp Activities, headed by Mr. Fosdick, which has been of great assistance to the Army in the intensive campaign of education to promote venereal prevention both among the soldiers and the civil population of the extra-cantonment zones in the United States.

While much has been accomplished in improving moral conditions in the Army, and among the civil population all over the United States, there is still far too low a standard of morality in civil and military life, and therefore a higher venereal rate in both than should exist in this age of enlightenment. However, the wonderful improvement in the morality of our soldiers should encourage all right-thinking men to strive and hope for even higher standards of living, not only in the Army, but among all the people of the United States.

Those who live in the past, " the old-timers ", are still wont to say : — " Young men are going to indulge in illicit sexual relations, and there is no use denying it, or trying to do anything to stop this age-long evil ". Fortunately this class of reactionaries is growing smaller, and the progressive element, both among the laity and the medical profession, knows that a great deal has been done in practical venereal prophylaxis that has been based upon scientific facts and practical " commonsense " methods. They also realize that much greater effort is needed to protect the men and women of our country against the diseases that make soldiers unfit for fighting, fill insane asylums, shorten life, decrease birthrates, and altogether cause more misery and poverty than any other class of diseases.

G. H. Q. Bulletin No. 54 shows that the United States Army authorities have a rational viewpoint regarding venereal diseases, and that they understand the underlying causes. This Bulletin is an evidence of the fact that they are making an earnest effort to improve moral conditions among our soldiers. It will be far-reaching in its effect upon the morals of the men in the Army; and its bearing upon the vice problem in civil life will be utilized for years to come in the anti-vice crusades in American cities, because it places the United States Army squarely on record as recognizing the dangers of " regulated and inspected houses of prostitution ".

### Venus and Bacchus

The relationship between alcohol and illicit sexual indulgence is recognized in G. H. Q. Bulletin No. 54 which says : " In the majority of cases drunkenness precedes and leads to exposure to venereal infection ". Apropos of this is the remark that is credited to Sir William Osler: "Man worships at the shrine of Bacchus early in the evening; a few hours later he is enamored of Venus; and then he becomes a devotee of Mercury for two years". Total abstinence from alcohol would seem to be the moral that should be drawn from these observations by such distinguished authorities. It is just as well to state facts and say that the wisest course for the American Army officer or the private soldier of the American Expeditionary Forces to pursue is to abstain from wine and all other forms of alcoholic beverages, if he would resist the temptations that surround him and go back home "clean" and free from diseases which may not only interfere with his own happiness and usefulness, but which may destroy the health, and even the life, of the woman whom he will marry in the years to come, or the one whom he has already vowed to honor and protect.

In the early months of the war, American soldiers embarking from New York were granted permissions of one or two days to see sights of America's greatest city. Venereal inspection was held before embarkation, and those soldiers infected were not allowed to sail. So many of them contracted venereal diseases in New York, which developed before or soon after their arrival in France, that the leave privilege was taken away from the soldiers passing through that port. There was a marked reduction in venereal diseases following this action of military expediency, which many thought a hardship, but which increased the number of soldiers available for fighting. Experience at the ports of France was the same as in New York. (The Medical Bulletin, published by the American Red Cross, May 1918, Vol. 1, No. 7. pp. 497-498).

Observations regarding the incidence of venereal diseases among the American soldiers, particularly officers, has shown that Paris has been the source of infection for many men. On one occasion, nine officers spent one night in Paris. Seven of them contracted syphilis. A group of 45 soldiers spent 6 hours in Paris and 9 of them became infected with venereal diseases. These and many other similar incidents are vouched for by reliable medical officers. Men who become intoxicated neglect venereal prophylaxis and the strangers in cities do not know where prophylactic stations may be found. It is a fact that in Paris, where young and sometimes older men "relax" after their arduous duties at the front or in offices, the venereal rate is disgracefully high. These facts should be known to the men who visit this beautiful city. They should also be made to realize that in running unnecessary risks they may be participes criminis in depriving others of the privilege of enjoying a visit to one of the most historic, as well as one of the most beautiful, cities of the world.

### The Responsibility of Company Commanders.

Venereal prophylaxis is in reality as much of a problem for the line officers as for medical men. If the commanding officers of various units lead clean, sober lives and enforce the army regulations, there will be a low venereal rate among the men under them. If they have loose morals and are lax in discipline, they will have high non-effective rates from this class of preventable disease. Illustrating this is an incident that occurred in one of the smaller cities of France. The number of men on "sick report" in one company of a certain regiment was much higher than that of other units in the same organization. When the report reached the Division Commander he sent out through regular military channels an enquiry as to the causes for this high venereal rate in this company. The Battalion Commander returned the letter endorsed as follows : "The cause of the high venereal rate in Company ... has been discovered and the remedy applied. Captain Z has been removed and Captain A has been placed in charge. Company ... will soon have a low non-effective rate from venereal disease". Subsequent reports showed that the venereal rate of a company depends largely upon the morals and discipline of its captain. To a great extent the responsibility of the morals of a unit rests with the commanding officer, and he should be held accountable for the loose discipline resulting in a high venereal rate among the men in his command.

### Venereal Diseases and Birth Rates

The American soldiers (officers and privates) who have come to France represent the highest type of manhood in our country. They will be the fathers of the next generation of men and women upon whom great responsibilities will fall; and they must make up for the loss of the gallant soldiers who have died in the service of their country in this war. American soldiers therefore have no right to run the risk of becoming infected with diseases that may reduce our birth-rate, or which may be transmitted to the children with whom it is their duty to repopulate the land of their birth and pride.

The French regard their low birth rate as one of their most serious problems, and they fear a further reduction from the increase in venereal diseases that has occurred in the past four years. Thibierge, in his book "*Syphilis et l'Armée*" published by Masson et Cie, Paris, 1917, quoting from Pantries, says "If one admits the very probable number of 200,000 cases of syphilis (in the French Army) and attributes to each of these cases the production of only two abortions, he sees that syphilitic infections will cost 400,000 births or the equivalent of two yearly classes of soldiers". This does not include the sterility due to salpingitis and orchitis from gonorrheal infection.

### Education the Remedy.

Medical officers in the American Expeditionary Forces should impart these facts, and others which they know regarding venereal diseases, to every soldier in each branch of the service. The same kind of a campaign of education should be carried on at home until every American man and boy has learned the truth, that the prostitute, whether registered or clandestine, either in France or in the United States, almost surely has syphilis or gonorrhoea, or both; and that even though she may be a paragon of physical beauty, she is more dangerous to him than contact with a case of smallpox.

"Sexual continence is the plain duty of members of the A. E. F., both for the vigorous conduct of the war and for the clean health of the American people after the War". These are the plain and forceful words which the G. H. Q. Bulletin No. 54 uses. But, if men must give way to their sexual desires, this Bulletin says that they should follow the regulations regarding prophylaxis, which, while not an absolute specific in preventing venereal diseases, has done much to keep down the incidence of venereal diseases in the Army.

G. H. Q. Bulletin No. 54 deserves, and no doubt will go down in history as one of the greatest military documents ever written. Its high moral tone makes it the kind of advice that a father would like to send to his son, and it will be so used in this and future generations. Every man in the Army should be provided with a personal copy of this Bulletin, and he should also be given an extra copy to send to a son or brother back home. We consider it of such value as a contribution to preventive medicine that it is reproduced in the editorial columns of *War Medicine*.

### The Moral Side.

Now that peace has been declared and the work and tension of war are relaxed, it will be more difficult to maintain discipline among soldiers, many of whom still have the old idea that a good time, and "seeing the town" consists of debauchery with wine and women. It is difficult for them to realize that one such night may result, ten, twenty or thirty years later, in apoplexy, nephritis, paresis or other form of insanity, and many other conditions which cut men off in the prime of life; but every doctor knows of many such cases.

Now is the time to read G. H. Q. Bulletin No. 54 to line officers and men, because it not only points out the dangers of illicit sexual indulgence, but it appeals to the higher sense of right and to the patriotism of the splendid young men who are in the Armies of the American Expeditionary Forces. Medical officers, in performing their duty as instructors of personal hygiene in the army, should teach the soldiers in plain words the scientific facts regarding venereal disease and its epidemiology, and they should also call attention to the degrading effects of associating and cohabiting with immoral women, whether prostitutes or not.

Burns, though he did not always " reck the reed " of his own wisdom, perhaps understood the emotions of men, or what is called " human nature ", as well as any other poet. In his " Lines to a Young Friend " he brings out a thought which is worth while passing on to many young men now in France, who are away from the restraining influences of home at a time in life when they most need the advice of their fathers.

" The sacred lowe o' well placed love
Luxuriantly indulge it.
But never tempt the illicit rove,
Though nothing should divulge it.
I wave the quantum o' the sin,
The hazard o' concealing.
But ach ! It hardens a' within,
And petrifies the feeling. "

### G. H. Q.

AMERICAN EXPEDITIONARY FORCES.

BULLETIN No. 54. FRANCE, *Aug. 7, 1918.*

1. The disturbance of normal social conditions caused by war tends to a breakdown of moral standards and an increase of immorality and venereal disease. Verified statistics of actual experience in the present war show that a great danger of venereal infection confronts both the civil population and the army.

2. To combat this danger full dissemination of the facts about venereal disease and rigid enforcement of regulations are essential.

3. Attention of all members of the A. E. F. is directed to the information and regulations governing the prevention of venereal disease contained in G. O. Nos. 6, 34 and 77, 1917, and in this order. All officers will see that these regulations are completely understood and carried out throughout their commands. Failure in this will be serious evidence of inefficiency.

(A) *FACTS ABOUT VENEREAL DISEASE.*

The greatest source of venereal infection is the "regulated and inspected" house of prostitution. The methods of inspection are grossly ineffective. The women in these resorts are *not* free from infection. They frequently stay daily with a score or more of men, each thus passing the infection from one man to those following him. There are numerous cases of soldiers contracting both syphilis and gonorrhea at these houses. The placing of "regulated" houses of prostitution "off limits" at one seaport reduced venereal infection to one-eighth the previous rate.

Venereal infection is highly prevalent among unregistered "clandestine" prostitutes, and exists to-day to an increasing degree in social classes hitherto little suspected. The practice of illicit indulgence in sexual intercourse will almost inevitably lead to venereal infection sooner or later.

In the majority of cases drunkenness precedes and leads to exposure to venereal infection.

Failure to submit to prompt prophylaxis increases the percentage of incapacitating infection. The effectiveness of prophylaxis depends upon the promptness with which it is employed. Within the first hour the failures are only one-tenth of 1 per cent., second hour one-half of 1 per cent., and after three hours from 1½ to 7 per cent. The average rate of failure for the A. E. F. of 2 per cent. indicates that in many organizations the *prompt* submission to prophylaxis is not enforced.

[BUL. 54,]

The methods of regulation adapted under the general orders referred to above have steadily reduced the venereal rate from 84 new cases per thousand men per year in 1916 to 29 in the A. E. F. to-day.

The contraction of venereal disease incapacitates for service and often produces permanent impairment of health. It is a breach of duty to the country, army and fellow soldier.

(B) *CONTINENCE.*

Sexual continence is the plain duty of members of the A. E. F., both for the vigorous conduct of the war and for the clean health of the American people after the war. Sexual intercourse is *not* necessary for good health, and complete continence is wholly possible. Careful studies show that only a relatively small proportion of members of the A. E. F. habitually indulge in sexual intercourse.

Commanding officers will urge continence on all men of their commands as their duty as soldiers and the best training for the enforced sexual abstinence at the front. Instruction, work, drill, athletics and amusements will be used to the fullest extent in furthering the practice of continence.

(C) *LEAVES.*

All night and week-end leaves are a fertile source of infection, multiplying contacts and delaying prophylaxis. Such leaves will be denied as much as possible.

(D) *DRUNKENNESS.*

The provisions of existing orders relating to the sale of intoxicants to members of the A. E. F. will be uniformly and strictly enforced. Cases of drunkenness will be dealt with by prompt disciplinary action.

(E) *PROPHYLAXIS.*

All means will be adopted to enforce the uniform and *early* use of prophylaxis.

(F) *COURTS-MARTIAL.*

Courts-martial will be sufficiently severe in dealing with cases of venereal infection to deter men from wilful exposure. The records of all sentences imposed will be carefully examined and compared, and lax courts and officers held strictly accountable.

(G) *TREATMENT.*

The importance of early treatment is so great that officers will urge their men to report for examination on any suspicion of disease.

(H) *HOUSES OF PROSTITUTION.*

Throughout the A. E. F. all houses of prostitution, as well as saloons indulging in the improper sale of intoxicants to members of the A. E. F., will be designated as " off limits. " Commanding officers will adopt the necessary means and disciplinary measures to prevent soldiers from visiting them.

[Bul. 54.]

(I) *APPREHENSION OF CLANDESTINE PROSTITUTES.*

By co-operation with the French police, military and civil authorities, every effort will be made to repress clandestine prostitution and street walkers and employ every available means under the French law to have all such women sent away.

(J) *REPORTS.*

Reports of conditions in contravention of the purposes of this order will be made by military police and all officers concerned.

4. The C. in C. enjoins upon all members of the A. E. F. the strictest observance of sexual continence. His position on this question is stated, as follows, in a letter appointing representatives to a British-American Conference on the subject :

"I have heard with great satisfaction of the recent decision of the British War Office that the licensed houses of prostitution are to be put out of bounds in the B. E. F. Many of us who have experimented with licensed prostitution or kindred measures, hoping thereby to minimize the physical evils, have been forced to the conclusion that . . . . . abolition as distinguished from regulation is the only effective mode of combatting this age-long evil. I have the greatest hope that the results of the conference which you have called will be far-reaching in their effect. This menace to the young manhood in the army forces and to the health and future well-being of our peoples cannot be met by the efforts of each Government working apart from the others . . . . . . . The gravest responsibility rests on those to whom the parents of our soldiers have intrusted their sons to the battle, and we fail if we neglect any effort to safeguard them in every way.

"We have the common ground of humanity; we have the well considered conclusions of the best scientific minds on our side, and from the fact that, in this war of nations-in-arms, the soldier is merely a citizen on war service, we have all the elements which will force co-operation between military and civilian authorities. With our nations co-operating hand-in-hand, . . . . . we have the brightest prospects of winning the victory."

By command of General Pershing :

JAMES W. McANDREW,
*Chief of Staff.*

Official :

ROBERT C. DAVIS,
*Adjutant General:*

A. G. Printing Dept., G. H. Q. A. E. F., 1918.

